# Injury characteristics and predictors of mortality in patients undergoing pancreatic excision after abdominal trauma: A National Trauma Data Bank (NTDB) study

**DOI:** 10.1097/MD.0000000000033916

**Published:** 2023-06-16

**Authors:** Nasser A.N. Alzerwi

**Affiliations:** a Department of Surgery, College of Medicine, Majmaah University, Ministry of Education, AL-Majmaah City, Riyadh Region, Kingdom of Saudi Arabia.

**Keywords:** injury mechanism, mortality, pancreatic excision, splenectomy, trauma

## Abstract

Pancreatic tumors and pancreatitis are the main indications for pancreatic excision (PE). However, little is known about this type of intervention in the context of traumatic injuries. Surgical care for traumatic pancreatic injuries is challenging because of the location of the organ and the lack of information on trauma mechanisms, vital signs, hospital deposition characteristics, and associated injuries. This study examined the demographics, vital signs, associated injuries, clinical outcomes, and predictors of in-hospital mortality in patients with abdominal trauma who had undergone PE. Following the Strengthening the Reporting of Observational Studies in Epidemiology guidelines, we analyzed the National Trauma Data Bank and identified patients who underwent PE for penetrating or blunt trauma after an abdominal injury. Patients with significant injuries in other regions (abbreviated injury scale score ≥ 2) were excluded. Of the 403 patients who underwent PE, 232 had penetrating trauma (PT), and 171 had blunt trauma (BT). The concomitant splenic injury was more prevalent in the BT group; however, the frequency of splenectomy was comparable between groups. In particular, concomitant kidney, small intestine, stomach, colon, and liver injuries were more common in the PT group (all *P* < .05). Most injuries were observed in the pancreatic body and tail regions. The trauma mechanisms also differed between the groups, with motor vehicles accounting for most of the injuries in the BT group and gunshots accounting for most of the injuries in the PT group. In the PT group, major liver lacerations were approximately 3 times more common (*P* < .001). The in-hospital mortality rate was 12.4%, with no major differences between the PT and BT groups. Furthermore, there was no difference between BT and PT with respect to the location of the injuries in the pancreas, with the pancreatic tail and body accounting for almost 65% of injuries. Systolic blood pressure, Glasgow Coma Scale score, age, and major liver laceration were revealed by logistic regression as independent predictors of mortality, although trauma mechanisms and intent were not linked to mortality risk.

## 1. Introduction

Pancreatic injuries present a complex challenge for trauma surgeons.^[1]^ The pancreas is an important gland that secretes several hormonal and digestive enzymes to maintain normal body functions.^[2]^ Insulin and glucagon, 2 hormones released by the pancreas, are vital for glucose metabolism. The pancreas also influences nutritional health.^[3,4]^ Pancreatic injuries and pancreatic excision (PE) as a treatment modality can adversely affect endocrine and exocrine pancreatic function.^[2,5]^

The reported morbidity rate for pancreatic injuries is approximately 45%.^[6,7]^ Surgical procedures for the pancreas include excision, partial pancreatectomy, and total pancreatectomy. Pancreatic excision involves the removal of a small lesion, whereas partial pancreatectomy removes a specific portion of the pancreas. Total pancreatectomy is performed in patients with severe chronic pancreatitis or extensive pancreatic cancer. The choice of surgery depends on the patient’s condition and the extent of the pancreatic lesion. Pancreatectomy requires careful consideration due to the proximity of the pancreas to critical abdominal organs and related morbidity concerns. Although many treatment techniques are available, the superiority of 1 modality over the other has not been convincingly demonstrated, especially in the context of abdominal trauma. Pancreatectomy procedures fall into 2 broad categories: those that remove the entire pancreas and those that remove a segment of the pancreas. Managing blood sugar levels after a total pancreatectomy is difficult because of the loss of pancreatic endocrine and exocrine function.^[8]^ However, partial pancreatectomy (PP) preserves endocrine and exocrine functions, is a proven surgery for injuries of the pancreatic tail and body,^[9]^ and is typically used in approximately 13% of patients undergoing pancreatic trauma treatment.^[10]^ Consequently, unless there are exceptional circumstances, PP is performed more often than total pancreatectomy.^[11]^

However, PP is not completely free of endocrine complications. The prevalence of diabetes was 16% greater in patients with PP than in the general population.^[12]^ In another study, approximately 18% of the patients had new-onset post-PP diabetes mellitus.^[13]^ Additionally, PP may have a long-term detrimental effect on dietary health, cardiorespiratory fitness, and well-being; hence, knowing the intent and mechanism of trauma and the outcome and other related factors and outcomes is crucial in patients who receive PP after a traumatic injury.^[14,15]^

The National Trauma Data Bank (NTDB) is the largest repository for traumatic injuries.^[16–28]^ Using the NTDB, Phillips et al analyzed data from 777 patients with penetrating injuries to determine mortality, injury patterns, and predictors of prognosis; however, they did not focus on PP. Similarly, another database study covering the 2008 to 2010 NTDB was published in 2014 and reported that pancreaticoduodenectomy did not improve outcomes.^[29]^ While Kuza et al (2020)^[30]^ conducted a recent study on pancreatic injuries in abdominal trauma utilizing data from the NTDB between 2007 and 2011 and reported an overall morbidity rate of 53% and mortality rate of 21.2%, insights into the patterns of traumatic injuries and the mechanisms of pancreatic resection, as well as the associated mortality and complications in trauma patients after pancreatic resection, remain limited. Therefore, it is imperative to conduct further research to comprehensively understand the factors contributing to these outcomes and optimize the management of pancreatic injuries in trauma patients, given the significant clinical and economic burden associated with such injuries.

This is the first NTDB study to assess PE in individuals with blunt (BT) or penetrating traumas (PT). The main goals of this study were to analyze the use of PEs in abdominal trauma, identify injury-related characteristics, assess morbidity and mortality rates, and investigate predictors of mortality.

## 2. Methods

We queried the NTDB for 2017 for trauma patients who had undergone proximal pancreatectomy, radical subtotal pancreatectomy, distal pancreatectomy, and other PP after abdominal and pelvic trauma as defined by the abbreviated injury scale. Cases of injuries to other abbreviated injury scale regions were also disregarded.^[31–35]^ The Strengthening the Reporting of Observational Studies in Epidemiology guidelines were followed in this study. Patients with PE, as defined by the International Classification of Disease and Procedure Coding System (ICD-10-PCS Code:0FBG0ZZ), were included in further analysis. This study included all types of trauma and underlying mechanisms. Patient demographic data, pancreatic injury location, injury severity score (ISS), shock (systolic blood pressure (SBP) < 90 mm Hg), and total and motor Glasgow coma scale (GCS) scores.

In-hospital mortality rate was the primary outcome. The prevalence of pancreatic injury, length of hospital stay (LOS), serious complications, discharge disposition, intensive care unit LOS, and ventilator days were considered secondary outcomes. For uniformity, nomenclature from the NTDB database was maintained. The mechanism of injury was defined under 6 categories: motor vehicle accidents (MVA), fall, struck, stab (cut/pierce), gunshot wound (GSW), firearms, and others.^[36]^

### 2.1. Statistical analysis

To report continuous data, the median and interquartile range were used, whereas the frequency (%) was used to describe categorical variables. Incomplete or missing patient data were not included in this study. Pearson chi-square or Fisher exact tests were used for categorical data, while Kruskal–Wallis and Mann-Whitney *U* tests were conducted for continuous data. Univariate and multivariate logistic regression analyses were used to identify the factors associated with in-hospital mortality. Statistical significance was set at *P* < .05.

## 3. Results

### 3.1. Demographics and clinical attributes

**Figure 1.**
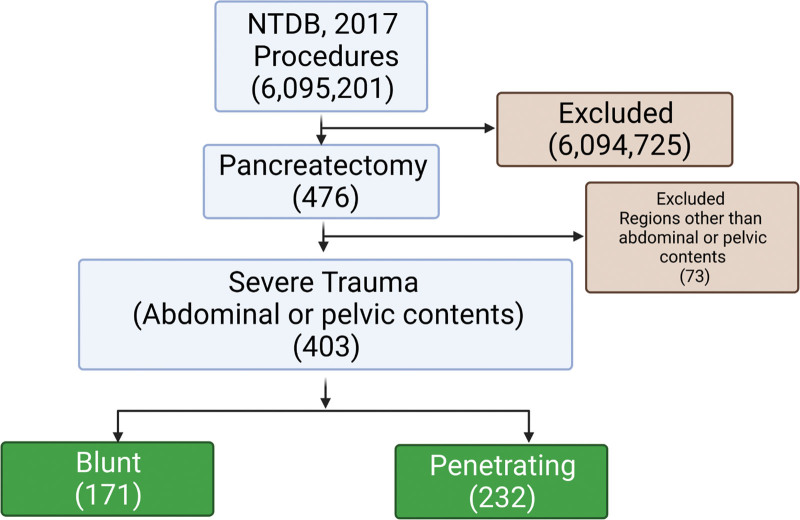
Study flowchart representing exclusion and inclusion of patients.

More than 6 million procedures were conducted in 2017; PE was performed in 476 patients, and 403 patients were included after eliminating other injured areas, except for the abdominal and pelvic regions (Fig. 1 and Table 1). Patients who underwent PE were more likely to be men (78.7%) (*P* < .001). The age of the patients was 29.0 (20.0, 45.0) years. SBP, GCS (MOTOR), GCS, and ISS were 112.0 (92.0, 132.0), 6.0 (5.0, 6.0), 15.0 (12.0, 15.0), and 26.0 (18.0, 34.0) respectively. In terms of location on the pancreas, most had an injury on the tail (39.2%). The organs most frequently observed to be associated with injury were the spleen (59.1%), gallbladder (42.7%), liver (42.7%), and kidneys (41.2%). The most frequent mechanism was GSW, observed in 212 patients (52.6 %), followed by MVA in 101 patients (25.1 %). Assault (197, 48.9%) was the most common intent, followed by unintentional (169, 41.9%) (Table 1).

**Table 1:** Demographics, clinical features, and trauma mechanisms in patients with PE.

Median (Q1, Q3) or N (%)	BT (N = 171)	PT (N = 232)	Total (N = 403)	*P* value
Age (yr)	31.0 (20.0, 51.0)	28.0 (21.0, 41.0)	29.0 (20.0, 45.0)	.413
Age > 60 yr	17 (9.9%)	9 (3.9%)	26 (6.5%)	.014
Sex (Men)	112 (65.5%)	205 (88.4%)	317 (78.7%)	<.001
ISS	27.0 (17.0, 38.0)	26.0 (19.0, 34.0)	26.0 (18.0, 34.0)	.741
ISS > 15	142 (83.0%)	203 (87.5%)	345 (85.6%)	.208
SBP	114.0 (97.0, 130.5)	110.0 (90.0, 134.0)	112.0 (92.0, 132.0)	.241
SBP < 90	27 (15.8%)	51 (22.0%)	78 (19.4%)	.120
GCS (motor)	6.0 (6.0, 6.0)	6.0 (5.0, 6.0)	6.0 (5.0, 6.0)	.564
GCS	15.0 (12.0, 15.0)	15.0 (12.0, 15.0)	15.0 (12.0, 15.0)	.573
GCS < 8	33 (19.3%)	45 (19.4%)	78 (19.4%)	.980
Race				
** White**	114 (66.7%)	62 (26.7%)	176 (43.7%)	<.001
** Black**	30 (17.5%)	133 (57.3%)	163 (40.4%)	<.001
** American Indian**	3 (1.8%)	4 (1.7%)	7 (1.7%)	.982
** Asian**	5 (2.9%)	2 (0.9%)	7 (1.7%)	.117
** Other**	14 (8.2%)	23 (9.9%)	37 (9.2%)	.553
Ethnicity				.042
** Hispanic or Latino**	31 (18.7%)	25 (11.3%)	56 (14.5%)	
** Not Hispanic or Latino**	135 (81.3%)	196 (88.7%)	331 (85.5%)	
The injured part of the pancreas				
** Head**	12 (7.0%)	21 (9.1%)	33 (8.2%)	.462
** Body**	37 (21.6%)	61 (26.3%)	98 (24.3%)	.282
** Tail**	70 (40.9%)	88 (37.9%)	158 (39.2%)	.541
** Unspecified**	42 (24.6%)	72 (31.0%)	114 (28.3%)	.154
Associate injury				
** Spleen**	113 (66.1%)	125 (53.9%)	238 (59.1%)	.014
** Gallbladder**	60 (35.1%)	112 (48.3%)	172 (42.7%)	.008
** Stomach**	13 (7.6%)	119 (51.3%)	132 (32.8%)	<.001
** Small bowel**	34 (19.9%)	76 (32.8%)	110 (27.3%)	.004
** Colon**	31 (18.1%)	92 (39.7%)	123 (30.5%)	<.001
** Kidney**	38 (22.2%)	128 (55.2%)	166 (41.2%)	<.001
** Bladder**	2 (1.2%)	6 (2.6%)	8 (2.0%)	.314
** liver**	60 (35.1%)	112 (48.3%)	172 (42.7%)	.008
** Major laceration liver**	13 (7.6%)	54 (23.3%)	67 (16.6%)	<.001
AIS severity				.053
** 2**	11 (6.4%)	14 (6.0%)	25 (6.2%)	
** 3**	50 (29.2%)	39 (16.8%)	89 (22.1%)	
** 4**	49 (28.7%)	81 (34.9%)	130 (32.3%)	
** 5**	56 (32.7%)	93 (40.1%)	149 (37.0%)	
** 6**	1 (0.6%)	0 (0.0%)	1 (0.2%)	
** 9**	4 (2.3%)	5 (2.2%)	9 (2.2%)	
Mechanism				<.001
** MVA**	101 (59.1%)	0 (0.0%)	101 (25.1%)	
** Fall**	12 (7.0%)	0 (0.0%)	12 (3.0%)	
** Struck**	14 (8.2%)	0 (0.0%)	14 (3.5%)	
** Stab**	0 (0.0%)	20 (8.6%)	20 (5.0%)	
** GSW**	0 (0.0%)	212 (91.4%)	212 (52.6%)	
** Other**	44 (25.7%)	0 (0.0%)	44 (10.9%)	
Intent				<.001
** Unintentional**	159 (93.0%)	10 (4.3%)	169 (41.9%)	
** Self-inflicted**	3 (1.8%)	20 (8.6%)	23 (5.7%)	
** Assault**	9 (5.3%)	188 (81.0%)	197 (48.9%)	
** Undetermined**	0 (0.0%)	5 (2.2%)	5 (1.2%)	
** Other**	0 (0.0%)	9 (3.9%)	9 (2.2%)	

AIS = abbreviated injury scale, BT = Blunt trauma, GCS = Glasgow coma scale, GSW = gunshot wound, ISS = injury severity score, MVA = motor vehicle accidents, PE = pancreatic excision, PT = penetrating trauma, SBP = systolic blood pressure.

### 3.2. Blunt and penetrating trauma

**Figure 2.**
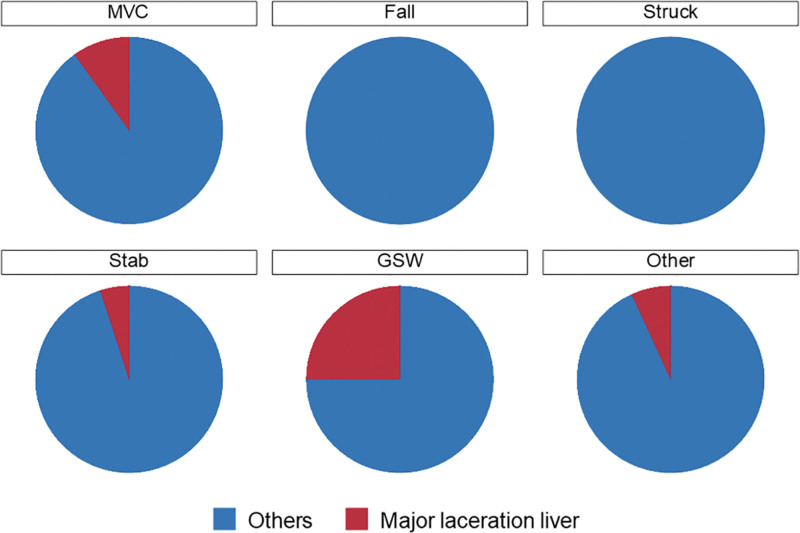
Trauma mechanism and liver laceration in abdominal trauma patients who underwent PE. PE = pancreatic excision.

Demographic and clinical characteristics were further stratified into the blunt and penetrating trauma groups (Table 1). PT was more common in men (PT, 88.4%; BT, 65.5%; *P* < .001), whereas the median SBP, ISS, and GCS did not differ significantly (All *P* > .05, Table 1). Notably, white patients were more common (66.7 %, *P* < .001) in the BT group, and black patients constituted the majority (57.3%, *P* < .001) in the PT group. The relative incidence of unintentional injury was significantly higher in BT (93.0%) than in PT (4.3%; *P* < .001). The trauma mechanism in 91.2 % of patients with PT was GSW (*P* < .001, Table 1). No discernible difference in pancreatic damage was evident, with both groups having the highest proportion of patients with tail injuries (*P* = .541). The organ most frequently observed to be associated with injury was the spleen, the incidence of which was significantly higher in the BT group (*P* = .014). In contrast, the gallbladder, liver, colon, and kidney injuries were higher in the PT group (All *P* < .05). The incidences of major lever lacerations were also higher in the PT group (BT:7.6% vs PT:23.3%, *P* < .001). The distribution of liver lacerations according to different injury mechanisms is shown in Figure 2.

### 3.3. BT and PT: outcome

Compared with BT, the PT group received significantly more blood transfusions during the first 4 hours (*P* < .001). The ventilator days and LOS in (intensive care unit) were similar for patients with PT and BT (*P* = .596); however, the LOS (the hospital) was higher in the PT group (*P* = .026). Cardiac arrest was noted in 9.1% and 3.1% of the patients in the PT and BT groups, respectively (*P* = .028). The PT group also had a higher risk of sepsis and readmission to the operating room (Table 2).

**Table 2:** In-hospital complications in abdominal trauma patients who underwent PE.

Median (Q1, Q3)	Blunt (N = 171)	Penetrating (N = 232)	Total (N = 403)	*P* value
Length of stay (d)	15.0 (8.0, 25.0)	18.0 (10.0, 29.5)	17.0 (9.0, 28.0)	.026
ICU d	6.0 (4.0, 13.0)	6.0 (4.0, 14.0)	6.0 (4.0, 13.0)	.596
Ventilator d	3.0 (2.0, 9.0)	4.0 (2.0, 8.0)	4.0 (2.0, 8.0)	.876
In-hospital complications				
Deep surgical site infection	5 (2.9%)	9 (3.9%)	14 (3.5%)	.605
Deep vein thrombosis (DVT)	6 (3.5%)	13 (5.6%)	19 (4.7%)	.327
Cardiac arrest with CPR	6 (3.5%)	21 (9.1%)	27 (6.7%)	.028
Extremity compartment syndrome	1 (0.6%)	1 (0.4%)	2 (0.5%)	.828
Unplanned intubation	8 (4.7%)	13 (5.6%)	21 (5.2%)	.680
Acute kidney injury	8 (4.7%)	17 (7.3%)	25 (6.2%)	.276
Myocardial infarction	1 (0.6%)	2 (0.9%)	3 (0.7%)	.749
Organ/space surgical site infection	5 (2.9%)	19 (8.2%)	24 (6.0%)	.027
Other	21 (12.3%)	23 (9.9%)	44 (10.9%)	.451
Acute respiratory distress syndrome (ARDS)	3 (1.8%)	7 (3.0%)	10 (2.5%)	.421
Unplanned return to the OR	12 (7.0%)	32 (13.8%)	44 (10.9%)	.031
Severe sepsis	3 (1.8%)	14 (6.0%)	17 (4.2%)	.035
Pressure ulcer	4 (2.3%)	6 (2.6%)	10 (2.5%)	.875
Unplanned admission to the ICU	15 (8.8%)	18 (7.8%)	33 (8.2%)	.714
Ventilator-associated pneumonia (VAP)	5 (2.9%)	2 (0.9%)	7 (1.7%)	.117
Mortality	17 (9.9%)	33 (14.2%)	50 (12.4%)	.197

ICU = intensive care unit, OR = odds ratio, PE = pancreatic excision.

As expected, all the patients underwent PP, and there were no cases of radical pancreatectomy. In the PT group, a significantly higher percentage of patients underwent kidney resection (BT: 5.8% vs PT: 25.0%; *P* < .001). Between the groups, the frequency of liver, duodenum, or small intestine resections or splenectomy did not vary (all *P* > .05, Table 3).

**Table 3:** Operative procedures in trauma patients who underwent PE.

	Blunt	Penetrating	Total	*P* value
	(N = 171)	(N = 232)	(N = 403)	
Pancreatic resection	--	--	--	.
Repair pancreas, open	3 (1.8%)	9 (3.9%)	12 (3.0%)	.215
Drainage of pancreas device, open	13 (7.6%)	12 (5.2%)	25 (6.2%)	.318
Resect small intestine, open	1 (0.6%)	3 (1.3%)	4 (1.0%)	.478
Repair small intestine, open	5 (2.9%)	17 (7.3%)	22 (5.5%)	.054
Repair duodenum, open	9 (5.3%)	20 (8.6%)	29 (7.2%)	.197
Resection duodenum, open	2 (1.2%)	2 (0.9%)	4 (1.0%)	.758
Resection spleen, open	114 (66.7%)	154 (66.4%)	268 (66.5%)	.952
Resection kidney, open	10 (5.8%)	58 (25.0%)	68 (16.9%)	<.001
Resection liver, open	0 (0.0%)	3 (1.3%)	3 (0.7%)	.136

PE = pancreatic excision.

### 3.4. In-hospital mortality

The hospital mortality rate was 9.9% in the BT and 14.2% in the PT (*P* = .197, Table 2). The clinical and demographic details of the deceased and the survivors are presented in Table 4. Victims in the deceased group were more likely to be men (deceased: 92.0 % vs survivor: 76.8%, *P* = .014). The deceased group was older and had lower SBP, lower GCS (motor) and GCS (total) scores, and a higher ISS (*P* < .001). There was no difference in trauma mechanism or intent (*P* > .05, Table 4). Regarding the location of the pancreatic injury, there was no significant difference between the deceased and survivors, with both having the maximum number of patients with tail injuries. Although substantial lever lacerations were more common in the deceased group than in the survivor group, the frequency of damage to the gallbladder, liver, colon, and kidneys was not substantially different (28.0% vs 15.0%, *P* = .021).

**Table 4:** A comparison of survivors and deceased.

Parameter Median (Q1, Q3)	Survivors (N = 353)	Deceased (N = 50)	Total (N = 403)	*P* value
Age (yr)	28.0 (20.0, 42.0)	44.0 (26.0, 53.0)	29.0 (20.0, 45.0)	<.001
ISS	26.0 (18.0, 34.0)	34.0 (25.0, 41.0)	26.0 (18.0, 34.0)	.001
SBP	114.0 (94.0, 132.0)	95.0 (60.0, 128.0)	112.0 (92.0, 132.0)	.001
GCS (Motor)	6.0 (6.0, 6.0)	3.0 (1.0, 6.0)	6.0 (5.0, 6.0)	<.001
GCS	15.0 (13.0, 15.0)	7.0 (3.0, 14.0)	15.0 (12.0, 15.0)	<.001
Sex (Male)	271 (76.8%)	46 (92.0%)	317 (78.7%)	.014
Race				
Asian	5 (1.4%)	2 (4.0%)	7 (1.7%)	.191
Other	32 (9.1%)	5 (10.0%)	37 (9.2%)	.830
American Indian	7 (2.0%)	0 (0.0%)	7 (1.7%)	.315
Black	148 (41.9%)	15 (30.0%)	163 (40.4%)	.108
White	152 (43.1%)	24 (48.0%)	176 (43.7%)	.510
Ethnicity				.636
Hispanic or Latino	50 (14.8%)	6 (12.2%)	56 (14.5%)	
Others	288 (85.2%)	43 (87.8%)	331 (85.5%)	
Injury location on the pancreas				
Head	28 (7.9%)	5 (10.0%)	33 (8.2%)	.618
Body	90 (25.5%)	8 (16.0%)	98 (24.3%)	.143
Tail	142 (40.2%)	16 (32.0%)	158 (39.2%)	.265
Unspecified	95 (26.9%)	19 (38.0%)	114 (28.3%)	.103
Associated injuries				
Spleen	205 (58.1%)	33 (66.0%)	238 (59.1%)	.286
Gallbladder	146 (41.4%)	26 (52.0%)	172 (42.7%)	.155
Stomach	110 (31.2%)	22 (44.0%)	132 (32.8%)	.070
Small bowel	88 (24.9%)	22 (44.0%)	110 (27.3%)	.005
Colon	106 (30.0%)	17 (34.0%)	123 (30.5%)	.568
Kidney	146 (41.4%)	20 (40.0%)	166 (41.2%)	.855
Bladder	7 (2.0%)	1 (2.0%)	8 (2.0%)	.994
liver	146 (41.4%)	26 (52.0%)	172 (42.7%)	.155
Major laceration liver	53 (15.0%)	14 (28.0%)	67 (16.6%)	.021
AIS severity				.077
2	23 (6.5%)	2 (4.0%)	25 (6.2%)	
3	82 (23.2%)	7 (14.0%)	89 (22.1%)	
4	111 (31.4%)	19 (38.0%)	130 (32.3%)	
5	129 (36.5%)	20 (40.0%)	149 (37.0%)	
6	0 (0.0%)	1 (2.0%)	1 (0.2%)	
9	8 (2.3%)	1 (2.0%)	9 (2.2%)	
Mechanism				.575
MVA	89 (25.2%)	12 (24.0%)	101 (25.1%)	
Fall	11 (3.1%)	1 (2.0%)	12 (3.0%)	
Struck	12 (3.4%)	2 (4.0%)	14 (3.5%)	
Stab	18 (5.1%)	2 (4.0%)	20 (5.0%)	
GSW	181 (51.3%)	31 (62.0%)	212 (52.6%)	
Other	42 (11.9%)	2 (4.0%)	44 (10.9%)	
Intent				.557
Unintentional	153 (43.3%)	16 (32.0%)	169 (41.9%)	
Self-inflicted	20 (5.7%)	3 (6.0%)	23 (5.7%)	
Assault	169 (47.9%)	28 (56.0%)	197 (48.9%)	
Undetermined	4 (1.1%)	1 (2.0%)	5 (1.2%)	
Others	7 (2.0%)	2 (4.0%)	9 (2.2%)	

AIS = abbreviated injury scale, BT = blunt trauma, GCS = Glasgow coma scale, GSW = gunshot wound, ICU = intensive care unit, ISS = injury severity score, LOS = length of stay, MVA = motor vehicle accidents, SBP = systolic blood pressure.

In general, duodenal resection was observed in 4% of the patients with PE; the frequency of these procedures was 0.6% in survivors and 4% (*P* = .022) in the deceased group (Table 5). Spleen resection was the predominant additional procedure in both groups. Kidney resection was performed in 15.9% of the survivors and 24.0% of the deceased (*P* = .151). Furthermore, the frequency of liver or small intestine resection varied (all *P* > .05).

**Table 5:** Operative procedures in deceased and survivors.

	Survivors	Deceased	Total	*P* value
	(N = 353)	(N = 50)	(N = 403)	
Repair pancreas open	11 (3.1%)	1 (2.0%)	12 (3.0%)	.664
Drainage of pancreas device Open	23 (6.5%)	2 (4.0%)	25 (6.2%)	.490
Resect small intestine open	4 (1.1%)	0 (0.0%)	4 (1.0%)	.449
Repair small intestine open	21 (5.9%)	1 (2.0%)	22 (5.5%)	.250
Repair duodenum open	25 (7.1%)	4 (8.0%)	29 (7.2%)	.814
Resection duodenum open	2 (0.6%)	2 (4.0%)	4 (1.0%)	.022
Resection spleen open	118 (33.4%)	17 (34.0%)	135 (33.5%)	.936
Resection kidney open	56 (15.9%)	12 (24.0%)	68 (16.9%)	.151
Resection liver open	2 (0.6%)	1 (2.0%)	3 (0.7%)	.270

For the possible risk factors for in-hospital mortality, Tables 6 and 7 show the findings of univariate and multivariate analyses, respectively. Univariate analysis revealed that sex, age, SBP, GCS, ISS, and intent were associated with the risk of in-hospital mortality. No effect of trauma type (BT or PT) or mechanism was observed, although small bowel resection and major laceration of the liver had a significant association. All factors determined to be significant in the univariate analysis were considered in the stepwise multivariate analysis. Sex (odds ratio [OR] 0.289, 95% confidence interval [CI]: 0.09–0.92, *P* = .036), Age (OR 1.04, 95% CI: 1.02–1.07, *P* < .001), SBP (OR 0.98, 95% CI: 0.98–0.99, *P* < .001), Total GCS (OR 0.86, 95% CI: 0.81–0.93, *P* < .001), ISS (OR 1.05, 95% CI: 1.02–1.08, *P* = .004), and the major laceration of the liver (OR 2.49, 95% CI: 1.07–5.8, *P* = .034) were associated with the risk of in-hospital mortality (Table 7). The area under the ROC curve for the model was 0.87 (Figure S1, Supplemental Digital Content, http://links.lww.com/MD/J55, Supplemental Content, presents ROC curve for the model). The Hosmer–Lemeshow test yielded a chi-square statistic of 7.69, suggesting a reasonably good fit of the logistic regression model with the data. The *P* value for the test was 0.4640.

**Table 6:** Univariate analysis of risk factors for mortality.

Variable	Univariate	
	OR 95% CI	*P* value
Sex (Women)	0.29 (0.10–0.82)	.020
Age (yr)	1.03 (1.01–1.05)	<.001
SBP	0.98 (0.97–0.99)	<.001
GCS	0.84 (0.79–0.89)	<.001
ISS	1.05 (1.02–1.07)	<.001
Intent		
Unintentional	Ref	.
Self-inflected	1.43 (0.38–5.36)	.592
Assault	1.58 (0.83–3.04)	.167
Undetermined	2.39 (0.25–22.70)	.448
Other	2.73 (0.52–14.28)	.234
Associated injuries		
Spleen	1.40 (0.75–2.61)	.288
Gallbladder	1.54 (0.85–2.78)	.157
Small bowel	2.37 (1.29–4.35)	.006
Colon	1.20 (0.64–2.25)	.569
Kidney	0.95 (0.52–1.73)	.855
Bladder	1.01 (0.12–8.37)	.994
liver	1.54 (0.85–2.78)	.157
Major laceration of liver	2.20 (1.11–4.36)	.024
Injury location on the pancreas		
Head	1.29 (0.47–3.51)	.619
Body	0.56 (0.25–1.23)	.148
Tail	0.267 (0.37–1.31)	.70
Unspecified	1.66 (0.90–3.09)	.106
Mechanism		
MVA	0.67 (0.08–5.70)	.717
Fall	1.24 (0.25–6.21)	.797
Struck	0.82 (0.17–4.00)	.810
Stab	1.27 (0.62–2.59)	.511
GSW	0.35 (0.35–1.65)	.186

CI = confidence interval, GCS = Glasgow coma scale, GSW = gunshot wound, ISS = injury severity scale, MVA **=** motor vehicle accidents, OR = odds ratio, SBP = systolic blood pressure (mm Hg).

**Table 7:** Multivariate logistic regression of in-hospital mortality.

Variable	OR (95% CI)	*P* value
Sex	.289 (.091–.92)	.036
Age (yr)	1.043 (1.02–1.068)	0
SBP	.985 (.975–.995)	.003
GCS	.864 (.806–.926)	0
ISS	1.047 (1.015–1.081)	.004
Small bowel injury	1.974 (.932–4.18)	.076
Major laceration of liver	2.492 (1.074–5.783)	.034

CI = confidence interval, GCS = Glasgow coma scale, ISS = injury severity scale, OR = odds ratio, SBP = systolic blood pressure (mm Hg).

## 4. Discussion

**Figure 3.**
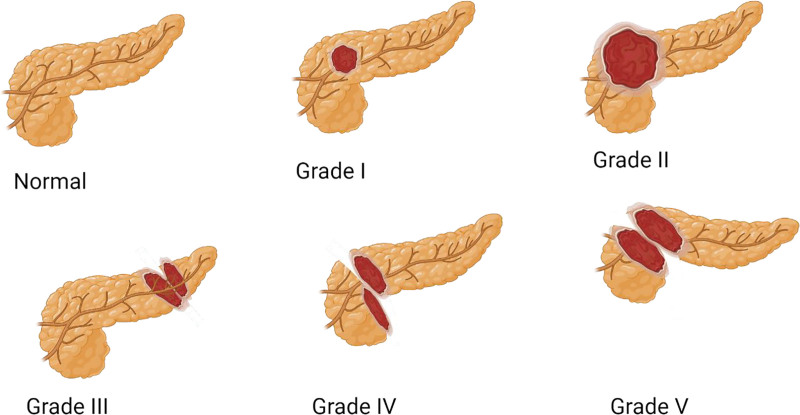
Pancreatic injuries are classified into 5 grades: Grade I (minor contusion or superficial laceration without duct injury), Grade II (major contusion or laceration without duct injury), Grade III (distal transection with duct injury), Grade IV (proximal transection), and Grade V (massive damage to the pancreatic head).

The pancreas can be affected by a wide range of traumatic injuries (Fig. 3). PP can lead to the deterioration of insulin secretion dynamics and glucose metabolism.^[4,37]^ To the best of our knowledge, this is the first NTDB analysis of adult patients with trauma requiring PP. Although the exact incidence of the injuries for which PP has been employed has not been documented in the literature, the previously reported incidence of pancreatic injury is 0.2% to 2% of all traumas and 0.2% to 12% of all abdominal trauma.^[5,6,15,30,38–42]^ In our study, the incidence of PP in trauma patients was 0.007% of all procedures performed in 2017; among these patients, 42% had BT, and the rest had PT. The frequency of pancreatic tail and body injuries was 39.2% and 24.3%, respectively. The distribution of injury location in the pancreas did not vary between BT and PT. The victims with PT were relatively younger, but the difference was not statistically significant.

There were considerably more male than female victims, and this difference increased even more in cases of PT, in which men represented almost 90% of all victims. In a remarkable 10-year multi-institutional study on pancreatic trauma, the average age was 30 years, and 70% of the victims were men.^[38]^ Consistent with our results, PT accounted for 63% of pancreatic injuries. Female trauma patients were much less likely to die than male trauma patients, confirming previous findings that there were sex differences in the manifestation of life-threatening sequelae post-trauma.^[43,44]^

The most prevalent causes of pancreatic injury were GSWs and MVAs. Previous studies have reported that MVAs are most common in BT, whereas GSWs are most common in penetrating traumas. Our analysis found that almost 59% of blunt pancreatic injuries were caused by MVA, whereas gunshot and stab (cut/pierce) wounds represent all-penetrating pancreatic trauma. Similar observations have been reported in other studies on US databases. Consistent with earlier data, men and younger patients seemed to make up most of those diagnosed with pancreatic injuries.^[30]^ Approximately 60% of the patients had concomitant spleen injuries, and approximately 40% had gallbladder, kidney, and liver injuries. Patients with non-abdominal injuries were excluded from the analysis. Approximately 17% of the patients exhibited major liver laceration, and this prevalence was found to be higher in patients with PT. Therefore, conducting a thorough search for additional abdominal lesions is crucial when examining pancreatic damage during diagnosis.^[30]^

We found that 12.4% of patients in our study died during hospitalization, with mortality rates of 9.9% and 14.2% for BT and PT, respectively. Complications occurred in several patients, and the rate was approximately the same in the BT and PT groups. Mortality rates of 11.5% were reported by Coyne et al^[41]^, who analyzed data from more than 25,000 subjects with pancreaticoduodenal damage. We found comparable mortality rates despite the broad range of patient demographics and population sizes. Kuza et al^[30]^ also analyzed a large group of patients with PT using the NTDB. In a study by Kuza et al, approximately 10% of patients had sepsis, with a greater frequency of PT, suggesting that the NTDB, although a diverse collection of trauma centers and other hospitals, likely sees more severe patients. Although the incidence of sepsis is higher in PT, it was found in only 4% of the patients in our population.

To determine the risk of in-hospital mortality, we used multivariate logistic regression to examine multiple factors, including associated liver, kidney, and spleen injuries. We found that major lacerations in the liver, age, GCS score, sex, and SBP were independently associated with in-hospital mortality. Coyne et al^[41]^ identified age and combined pancreaticoduodenal PT, injury, and surgery as the predictors of mortality. In another study, grade 5 pancreatic damage, advanced age, poorer GCS score, and greater heart rate were all found to be predictors of mortality in another study.^[45]^ Mortality was found to be associated with advanced age, ISS, shock, and nonoperative care in a UK study of almost 1000 patients who sustained the pancreaticoduodenal injury after trauma.^[46]^ Age, surgical intervention, and ISS were shown to correlate independently with mortality in an NTDB study of isolated blunt pancreatic injuries; however, the sex and severity of the pancreatic injury did not predict mortality.^[47]^ Although we limited the scope of our investigation to patients with PE, we discovered data that substantially confirmed past findings, emphasizing the importance of liver laceration.

Regarding the operative procedures used, duodenal resection was more prevalent in the deceased group, although the overall incidence was only 1%. Spleen resection was used in 66% of the cases; however, there was no difference in this procedure between the deceased and survivors. However, despite no distinction in trauma types, kidney resection was substantially more common in deceased patients. The frequency of other major procedures did not differ between the deceased and survivors or between BT and PT. There are multiple proposed treatment methodologies for pancreatic injury, with nonoperative management being prioritized for patients with good hemodynamic status and drainage, followed by exploration being prioritized for patients who are unstable and have sustained low-grade injuries.^[15]^ The current findings emphasize the need to avoid surgery, if feasible, with the caveat that damage control procedures may be essential in extreme cases.^[39,48]^ In particular, pancreatic resections are linked to increased mortality and lengthier hospitalizations.^[47,49]^ Importantly, ductal damage should be diagnosed during the first surgery, and surgical drainage should be initiated.^[50]^ Containment of abdominal trauma is a useful operational strategy for patients who are already physiologically exhausted and have multiple injuries.^[51]^ Finally, considering the complexities associated with pancreatic surgeries, patient outcomes after PE may be influenced by the competence and ability of the trauma surgeon.^[52]^

While the NTDB includes data from more than 900 hospitals, this information is not without limitations. Due to the retrospective nature of the investigation, we were unable to compare our patients with a control group or account for any confounders that were not evaluated. The NTDB is not a representative community sample. Therefore, these findings are applicable only to the types of hospitals that participated in the study. The NTDB may have missing data. Patients with associated injuries (such as the liver and spleen) were included in the study. As a result, the prognosis of individuals with PE may vary from that of individuals with hepatic injuries, even if both groups are already seriously ill. Surgical results and care depend greatly on the surgeon level of experience and training. Having no access to this data is also a limitation. Treatment options may be influenced by the monitoring of the patient’s hemodynamic conditions and intraoperative results. Consequently, we could not deduce whether surgery was a damage control strategy or predict when surgery would occur. Given that the NTDB uses ICD-10-CM procedure numbers to describe operations, it is probable that certain procedures have been under-reported. This study included data from 2017 only, which may have limited the sample size and generalizability of our conclusions. To enhance the robustness of our findings, there is a need for more comprehensive studies that encompass multiple years of data, which would allow for a larger sample size and a better representation of the population of interest. Furthermore, information on whether the procedures were performed in stages was unavailable; thus, we could not evaluate the patient’s age at death, the reason for death, or complications associated with surgery. All these are important considerations that should be included in further investigations with more current database information.^[42]^

## 5. Conclusion

The prevalence of PP among trauma patients was 0.007 %, with an associated mortality rate of 12.4 %. BT and PT involve considerably different mechanisms of trauma and clinical presentations. The organs most frequently observed to be associated with injury were the spleen, gallbladder, liver, and kidney. GSW was the most frequent injury mechanism, followed by the MVA. There were no statistically significant differences between the groups in terms of the site of pancreatic damage, with most patients having a pancreatic tail injury. There was no association between trauma type, intent, or mechanism and in-hospital mortality. However, sex, age, SBP, GCS score, and severe liver laceration were also associated with in-hospital mortality.

## Acknowledgements

The authors would like to thank all centers participating in the NTDB and all personnel responsible for reporting and maintaining the data in the registry. The authors thank the editors in www.editverse.com for their help in language editing and biorender.com, which has been used to prepare Figure 4. The authors would like to thank Deanship of Scientific Research at Majmaah University for supporting this work under Project Number No. 428.

## Author contributions

**Conceptualization:** Nasser Alzerwi.

**Data curation:** Nasser Alzerwi.

**Formal analysis:** Nasser Alzerwi.

**Funding acquisition:** Nasser Alzerwi.

**Investigation:** Nasser Alzerwi.

**Methodology:** Nasser Alzerwi.

**Project administration:** Nasser Alzerwi.

**Resources:** Nasser Alzerwi.

**Software:** Nasser Alzerwi.

**Supervision:** Nasser Alzerwi.

**Validation:** Nasser Alzerwi.

**Visualization:** Nasser Alzerwi.

**Writing – original draft:** Nasser Alzerwi.

**Writing – review & editing:** Nasser Alzerwi.

## Supplementary Material
