# Supplementary figures and images for: Injury characteristics and predictors of mortality in patients undergoing pancreatic excision after abdominal trauma: A National Trauma Data Bank (NTDB) study
Source: Medicine (Baltimore). 2023 Jun 16;102(24):e33916. doi: 10.1097/MD.0000000000033916 (PMC10270525; doi:10.1097/MD.0000000000033916)

## Supplementary Information

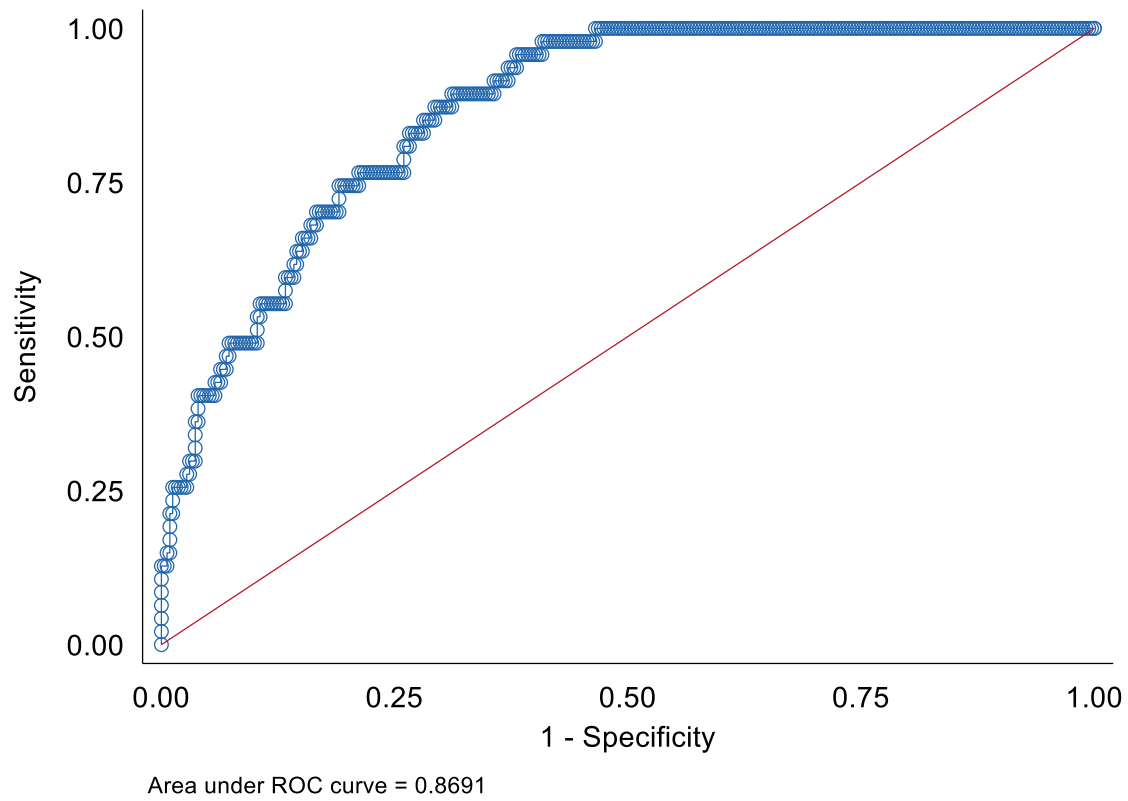

*Figure S1. ROC of multivariate analysis*

Supplement: Supplementary file 1 [file medi-102-e33916-s001.pdf]
